# Food Pesticide Residues Monitoring and Health Risk Assessment

**DOI:** 10.3390/foods13030474

**Published:** 2024-02-02

**Authors:** Yuwei Hua, Guangyang Liu

**Affiliations:** Institute of Vegetables and Flowers, Chinese Academy of Agricultural Sciences, State Key Laboratory of Vegetable Biological Breeding, Key Laboratory of Vegetables Quality and Safety Control, Laboratory of Quality & Safety Risk Assessment for Vegetable Products (Beijing), Ministry of Agriculture and Rural Affairs of China, Beijing 100081, China; 18434762011@163.com

This Special Issue presents a share of the work published in the journal *Foods* on pesticide residue monitoring and risk assessment in food. The topics presented herein included the pre-treatment of pesticide residues in food, detection methods for pesticide residues, and the risk assessment of pesticide residues [1,2]. These works targeting these themes are summarized below.

In order to accurately detect the levels of various kinds of pesticides across different food types, effective extraction, clean-up, and enrichment of the samples are imperative. Currently, the commonly used pretreatment techniques for pesticide residues include solid-phase extraction (SPE), solid-phase microextraction (SPME), magnetic solid-phase extraction (MSPE), matrix dispersive solid-phase extraction (MDSE), QuEChERS, gel permeation chromatography, molecularly imprinted solid-phase extraction (MISPE), and microwave-assisted extraction (MEA) [3,4]. Donghui Xu et al. (contribution 1), from the Institute of Vegetable and Flower Research of the Chinese Academy of Agricultural Sciences (CAAS), constructed a nanocomposite for magnetic solid-phase extraction of pyrethroids in tea beverages. Magnetic MWCNTs-ZIF-8 was functionalized by tetrabutylammonium chloride-dodecanol (DES5) to obtain a novel magnetic nanocomposite adsorbent, which can be used for solid-phase extraction of six pyrethroids in tea. The characterization results show that MM/ZIF-8@DES5 has a high specific surface area and superparamagnetism, which is conducive to the rapid enrichment of pyrethroids in tea beverage samples. The results of their optimization experiments indicate that DES5, consisting of tetrabutylammonium chloride and 1-dodecanol, was selected for the subsequent experiments and the adsorption performance of MM/ZIF-8@DES5 was higher than that of MM/ZIF-8 and M-MWCNTs. The validation results show that the method has a wide linear range (0.5~400 µg L^−1^, R^2^ ≥ 0.9905), a low detection limit (0.08~0.33 µg L^−1^), and good precision (intra-day RSD ≤ 5.6%, inter-day RSD ≤ 8.6%). The method was successfully applied to the determination of pyrethroid insecticides in three tea beverage samples and holds considerable promise for the monitoring of organic contaminants in environmental or food samples.

Traditional pesticide residue detection methods generally include gas chromatography–mass spectrometry (GC-MS), liquid chromatography–mass spectrometry (LC-MS), and high-performance liquid chromatography–mass spectrometry (HPLC-MS) [5]. These methods can accurately detect pesticides, but retain some limitations, such as complex and time-consuming pre-treatment steps, high training requirements for operators, expensive equipment, and inconvenience in terms of on-site analysis. In recent years, rapid detection has gradually become a popular research direction, and its application to the detection of pesticide residues can not only meet the needs of rapid, sensitive, and highly selective pesticide residue detection, but can also reduce the technical threshold for inspectors and the costs of testing [6,7]. Zhen Cao et al. (contribution 2), from the Institute of Agricultural Products Quality Standards and Testing Technology, CAAS, developed two rapid assays for the efficient determination of halosulfuron methyl (HM). High-quality anti-HM monoclonal antibody (Mab, No.1A91H11) was prepared by using a pyrazosulfuronamide of HM to generate semi-antigens and antigens. A direct competitive immunoassay (dcELISA) of Mab 1A91H11 was first established to obtain a half-maximal inhibitory concentration (IC50) of 1.5 × 10^−3^ mg/kg, with a linear range of 0.7 × 10^−3^ mg/kg–10.7 × 10^−3^ mg /kg. The sensitivity of the assay was shown to be 10 times higher than that of indirect competitive ELISA (icELISA). The average spiked recoveries were 78.9~87.9% and 103.0~107.4%, with coefficients of variation of 1.1~6.8% and 2.7~6.4% for tomato and corn substrates spiked with 0.01, 0.05, and 0.1 mg/kg HM, respectively. In addition, a magnetic lateral flow immunoassay (MLFIA) was developed for the quantitative detection of low concentrations of HM in rice water. The sensitivity of MLFIA was 3.3~50 times higher (IC50 of 0.21 × 10^−3^ mg/kg) than dcELISA. The average recovery of the developed MLFIA was 81.5~92.5%, with an RSD of 5.4~9.7%. Their results show that these two methods are suitable for the rapid detection of HM residues in substrate, corn substrate, and rice water, with improved sensitivity over traditional methods. Moreover, these two methods are very practical as a rapid test that is simple and easy to conduct. Fengnian Zhao et al. (contribution 3), from the School of Chemical and Materials Engineering, Beijing Technology and Business University (BTBU), constructed a portable three-electrode sensor based on laser-induced graphene (LIG) for the electrochemical detection of carbendazim (CBZ). The LIG three-electrode sensor can easily be produced by writing directly onto the PI film using a laser. The structure and composition of LIG were verified by SEM, Raman spectroscopy, and XPS, which confirmed its highly porous graphene structure and excellent specific surface area. The sensor’s detection performance was further improved by electrodepositing PtNPs onto the LIG surface. The prepared sensor (LIG/Pt) exhibited a wide linear range (1~40 µM), satisfactory LOD (0.67 µM), and good recovery (88.89~99.50%) in wastewater samples under optimal conditions. Furthermore, the electrochemical sensor is simple, sensitive, and selective, and provides a reliable real-time analytical method for the detection of CBZ residues in water samples. Fen Jin et al. (contribution 4), from the Institute of Agricultural Products Quality Standards and Testing Technology, Chinese Academy of Agricultural Sciences (CAAS), investigated the spatial distribution and migration characteristics of chloropyrifos, a plant growth regulator used for fruit pollination and fruit set, in fruit tissues. In this study, a matrix-assisted laser desorption/ionization mass spectrometry imaging (MALDI-MSI) method was developed for the first time to detect and quantify the dynamic results of clopyralid in fruits. The results show that clopyralid is mainly distributed in the pericarp and mesocarp portions of the fruit and shows a decreasing trend within 2 d of application. At the same time, the degradation rate of clopyralid was also detected using the HPLC-MS/MS method, and the results were similar to those resulting from the MALDI-MSI method, which proves that the results of detecting and quantifying clopyralid by this method are reliable. The establishment of this method provides a new means of pesticide detection and quantification, and the relevant residue results of chloropyrifuron provide data indicating the risks of its application.

Risk assessment refers to the scientific evaluation of biological, chemical, and physical hazards in food and food additives that may cause effects adverse for human health. Risk assessment is an invaluable basis for governments to formulate quality and safety standards and technical regulations for agricultural products, and it has become a necessary means for countries around the world to deal with technical barriers to trade in agricultural products and to regulate the import and export of agricultural products [8]. Zhan Dong et al. (contribution 5), from the Institute of Agricultural Products Quality Standard and Testing Technology, Shandong Academy of Agricultural Sciences, conducted the first comprehensive evaluation of the dissipation, metabolism, accumulation, handling, and risk assessment of flupyradifuran (FLU) and trichlormethrin (TRI) in cucumber and cowpea from cultivation to consumption. The results of the study show that flupyrine and TRI residues were higher in cowpea and dissipated more rapidly in cucumber. The major compounds found in the field samples were FLU and TRI (≤3256.29 µg/kg), while their metabolites, FLb and TRI, fluctuated at low residue levels (≤76.17 µg/kg). In addition, FLU and TRI accumulated in both cucumber and cowpea after repeated spraying, It was shown that peeling, washing, frying, boiling, and acid washing partially or largely removed FLU and TRI residues from raw cucumber and cowpea. In contrast, residues of the metabolite TRA were significantly enriched after acid washing. The risk assessments indicated that, based on the results of this study, chronic and acute exposure to both FLU and TRI through the consumption of cucumber and cowpea poses low health risks for children or adults. In the future, more experimental sites and crop categories should be selected nationwide to study the pathways of FLU and TRI residues to get a full picture of their actual dietary risks, which would be an important step in ensuring the safe use of FLU- and TRI-containing products and protecting human health. Yaohai Zhang et al. (contribution 6), from the Institute of Citrus Research, Southwest University, China, examined 573 kumquat samples originating from China for pesticide residues using the QuEChERS, UHPLC-MS/MS, and GC-MS/MS methods, which provided data for food safety checks of kumquats and enabled the reduction of human health risks. Their results show that 90% of the samples contained one or more pesticide residues. A total of 30 pesticides were detected, including 16 insecticides, 7 fungicides, 5 acaricides, and 2 plant growth regulators. Two of the pesticides had already been banned. The frequently detected pesticides included tebuconazole, spinosad, propiconazole, cyfluthrin, isoconazole, and imidacloprid. Two or more pesticide residues were found in 81% of the samples, and 9.4% of the samples had pesticide residues exceeding the MRLs, mainly including: isopropylphosphate, bifenthrin, triazophos, avermectin, thiocyclamfen, isoconazole, and thiram. Abamectin had the highest MRL exceedance rate at 1325%. The detection rate of the pesticides in kumquats was high and multiple residues were present, with about 81% of the qualified samples being contaminated. Ji-Ho Lee et al. (contribution 7), from the Department of Crop Science, Konkuk University, studied the dissipation kinetics of spirodiclofen and phenoxypyridinium 10 d after application, using pre-harvest time intervals. Spirodiclofen and phenoxypyrazone were applied in two greenhouses in Taean-gun, Chungcheongnam-do (Daejeon 1), and Gwangyang-si, Jeollanam-do (Daejeon 2), Republic of Korea. The samples were collected at 0, 1, 3, 5, 7, and 10 d after application. The method was validated using LCMS/MS, and the spiked recoveries were 82.0~115.9%. The biological half-lives of spirodiclofen and fenpropathrin were 4.4 and 3.8 d, respectively, in field 1, and 4.5 and 4.2 d, respectively, in field 2. The pre-harvest residue limits (PHRLs) of spirodiclofen on Aster were 37.6 mg/kg (field 1) and 41.2 mg/kg (field 2), respectively, and the PHRLs of fenpropathrin on Aster were 7.2 mg/kg (Field 1) and 3.6 mg/kg (Field 2), respectively. Hazard factors at pre-harvest intervals were broadly less than 100% for both pesticides—the exception being spirodiclofen at 0 days). Moreover, the HQs of spirodiclofen >100% and >25%, at day 0 and day 7 after application, respectively, could be considered risky.

Pesticide residue digestion is affected by a combination of environmental factors, pesticide endosorption, a variety of agricultural products, cultivation methods, soil quality, and other factors. The half-life and residue amount of pesticide residues varied in different experimental regions. Since different pesticide digestion prediction models are applicable to different backgrounds and have their own advantages and disadvantages, pesticide residue modeling combined with pesticide residue detection technology plays an important role in fitting the pesticide residue digestion law. Junsong Xiao et al. (contribution 8), from the College of Food and Hygiene, Beijing Technology and Business University (BTBU), investigated the effects of temperature and relative humidity on the degradation characteristics of five pesticides (carbendazim, fensulfuron, triadimefon, chlorpyrifos, and endosulfan) in wheat and flour, and developed a quantitative prediction model. Positive samples were prepared by spraying certain concentrations of the corresponding pesticide standards, and then storing the samples at different combinations of temperature (20 °C, 30 °C, 40 °C, 50 °C) and relative humidity (50%, 60%, 70%, 80%). The samples were collected at specific time points, ground, extracted, and purified for pesticide residue detection using the QuEChERS method, and then quantified by UPLC-MS/MS. Minitab 17 software was used to model the quantification of pesticide residues. The results show that high temperature and high humidity could accelerate the degradation of five pesticide residues, and the degradation curves and half-lives of different pesticides vary with temperature and relative humidity. A quantitative model of pesticide degradation in the whole process from wheat to flour was constructed, and the R^2^ of wheat and flour were greater than 0.817 and 0.796, respectively. This quantitative model can be used for the prediction of the pesticide residue levels in the process of wheat milling.

## Conclusions and Outlook

In terms of the current status of pesticide residues in food, differing levels of pesticide residues were detected in these studies, some of which were below the maximum residue limit values; however, some were at exceeded permitted levels and some banned pesticides were also detected. The results of risk assessment for pesticide residues in various types of food by the relevant organizations showed that some pesticides pose a dietary intake risk. What follows is a forecast of the possible directions the development of pesticide residue testing and risk assessment may take.

Pesticide Residue Detection Methods: (1) As the target range of pesticides increases, it is necessary to fine-tune detection conditions according to the characteristics of the food, to verify and optimize the established detection parameters to ensure that the detection methods are accurate and reliable, and to promote the development of the safe and standardized use and application of pesticides. (2) The maximum pesticide residue limits should be refined scientifically and effectively on the basis of risk assessment results and taking into account the biotoxicity of the pesticides, the existing levels of residues, people’s dietary intake, and other factors, to ensure that these limits are fully problem-oriented and that measures for residue management can be properly researched.

Pesticide Residue Risk Assessment: (1) Multi-methodology assessment—the currently widely adopted assessment models are limited to rough estimates of exposure and are unable to assess accumulation in target organs. Exposure assessment and early warning models can be improved to address this issue by exploring the use of relevant models from other fields. At present, many assessment methods applied in other industries have been introduced into pesticide residue risk assessment, which has led to a more diversified approach to food safety risk assessment. (2) Integrated assessment—if a particular hazardous factor in food is analyzed using only one risk assessment method, it is very vulnerable to subjectivity and methodological limitations. The risk assessment system should be brought into line with the characteristics of local populations and diets, and an assessment model should be constructed that aligns with the specific characteristics of different populations, ensuring a wider scope of application, fewer limitations, easy-to-obtain data, and greater accuracy. The simultaneous use of multiple assessment methods for comprehensive assessment will yield more comprehensive risk information.
